# Supplementary material for: A Novel DLG1 Variant in a Family with Brugada Syndrome: Clinical Characteristics and In Silico Analysis
Source: Genes (Basel). 2023 Feb 8;14(2):427. doi: 10.3390/genes14020427 (PMC9957379; doi:10.3390/genes14020427)
Supplement: Supplementary file 1 [file genes-14-00427-s001.zip › genes-2191873-supplementary.pdf]

genes

Supplementary data

Supplementary File

The Gene Panel Includes 132 Genes Associated with the Unexplained Sudden Death and GENE LIST BRUGADA SINDROME-V1.7.

ABCC9, ACTC1, ACTN2, AKAP9, ALMS1, ANK2, ANKRD1, BAG3, BRAF, CACNA1C, CACNA2D1, CACNB2, CALM1, CALM2, CALM3, CALR3, CASQ2, CAV3, CAVIN4, CHRM2, CRYAB, CSRP3, CTF1, CTNNA3, DES, DLG1, DMD, DOLK, DSC2, DSG2, DSP, DTNA, EMD, EYA4, FBN, FHL1, FKRP, FKTN, FLNC, GAA, GATA4, GATA5, GATA6, GATAD1, GJA5, GLA, GNB5, GPD1L, HCN4, HFE, HRAS, ILK, JPH2, JUP, KCNA5, KCND3, KCNE1, KCNE2, KCNE3, KCNE5, KCNH2, KCNJ2, KCNJ5, KCNJ8, KCNQ1, KRAS, LAMA4, LAMP2, LDB3, LMNA, LRRC10, LRP6, MAP2K1, MAP2K2, MIB1, MYBPC3, MYH6, MYH7, MYL2, MYL3, MYL4, MYLK2, MYOZ2, MYPN, NEBL, NEXN, NKX2-5, NRAS, PDLIM3, PKP2, PLN, PPA2, PRDM16, PRKAG2, PSEN1, PSEN2, PTPN11, RAF1, RANGRF, RIT1, RYR2, SCN10A, SCN1B, SCN2B, SCN3B, SCN4B, SCN5A, SGCD, SHOC2, SLC25A4, SLMAP, SNTA1, SOS1, TAZ, TBX20, TCAP, TECRL, TGFB3, TGFBR2, TMEM43, TMPO, TNNC1, TNNI3, TNNT2, TOR1AIP1, TPM1, TRDN, TRPM4, TTN, TTR, TXNRD2, VCL.

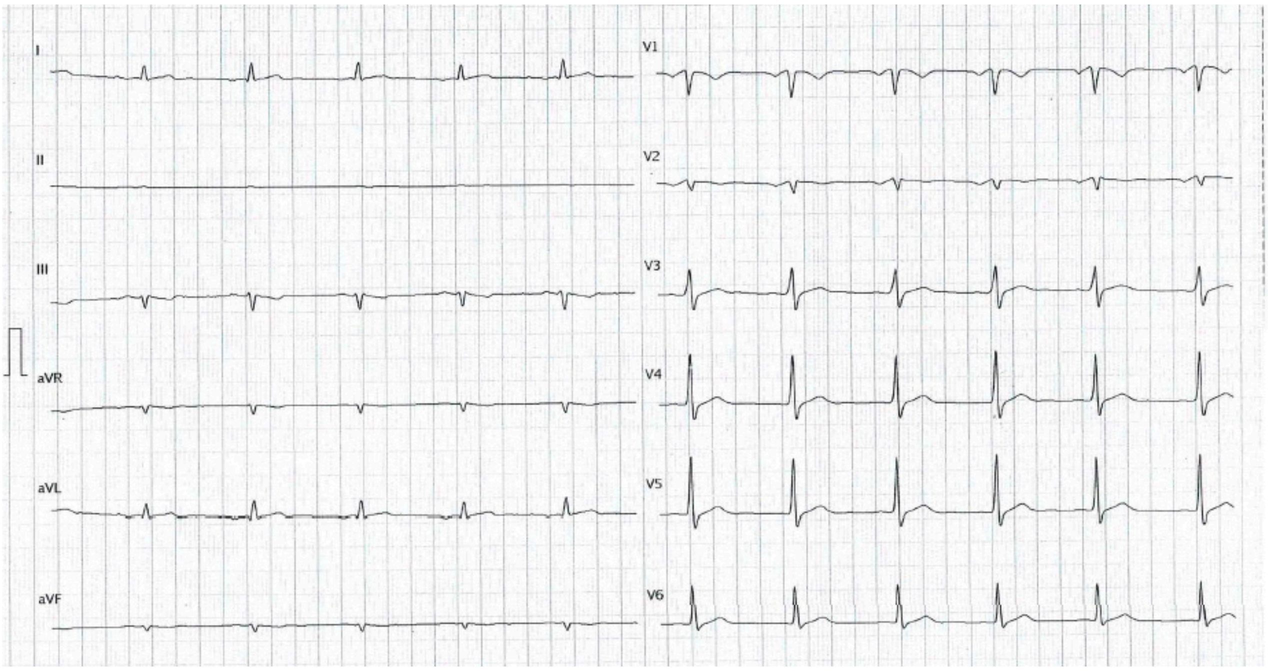

Figure S1. Rest 12-lead ECG of a family member with DLG1 mutation showing low QRS voltage in all peripheral leads (II-5).
